# Bias Due to Sample Selection in Propensity Score Matching for a Supportive Housing Program Evaluation in New York City

**DOI:** 10.1371/journal.pone.0109112

**Published:** 2014-10-13

**Authors:** Sungwoo Lim, Sue M. Marcus, Tejinder P. Singh, Tiffany G. Harris, Amber Levanon Seligson

**Affiliations:** 1 Bureau of Epidemiology Services, Division of Epidemiology, New York City Department of Health and Mental Hygiene, New York, New York, United States of America; 2 Department of Psychiatry and Biostatistics, Columbia University, New York, New York, United States of America; University of Pennsylvania, United States of America

## Abstract

**Objectives:**

Little is known about influences of sample selection on estimation in propensity score matching. The purpose of the study was to assess potential selection bias using one-to-one greedy matching versus optimal full matching as part of an evaluation of supportive housing in New York City (NYC).

**Study Design and Settings:**

Data came from administrative data for 2 groups of applicants who were eligible for an NYC supportive housing program in 2007–09, including chronically homeless adults with a substance use disorder and young adults aging out of foster care. We evaluated the 2 matching methods in their ability to balance covariates and represent the original population, and in how those methods affected outcomes related to Medicaid expenditures.

**Results:**

In the population with a substance use disorder, only optimal full matching performed well in balancing covariates, whereas both methods created representative populations. In the young adult population, both methods balanced covariates effectively, but only optimal full matching created representative populations. In the young adult population, the impact of the program on Medicaid expenditures was attenuated when one-to-one greedy matching was used, compared with optimal full matching.

**Conclusion:**

Given covariate balancing with both methods, attenuated program impacts in the young adult population indicated that one-to-one greedy matching introduced selection bias.

## Introduction

Propensity score matching has been widely used to reduce bias due to confounding in observational studies [1]–[3]. It allows researchers to examine distributions and differences in observed covariates between treatment (or exposed) and control groups using statistical and graphical tools, which is more advantageous for unbiased estimation than conventional regression adjustment that lacks such tools [4]. When addressing covariate imbalance via propensity score matching, optimal full matching has been shown to be more efficient than one-to-one greedy matching [5]. This is because optimal full matching minimizes the total distance between treatment and control groups, whereas one-to-one greedy matching performs localized matching in which a person in the treatment/exposed group is sequentially matched with a person in the control group [5], [6]. In addition, optimal full matching employs flexible matching ratios (e.g. N:N), which is more efficient in balancing covariates than matching restricted to one-to-one pairs [5], [7]. Along with improved internal validity via covariate balancing, optimal full matching can retain almost all subjects, unlike one-to-one greedy matching which only retains pairs of treatment and control subjects [5]. When evaluating public health interventions targeting certain populations, it is important to ensure comparability between the propensity score-matched population and the original population of interest. If they are systematically different due to the exclusion of unmatched subjects, then external validity (or generalizability) may be reduced [8], [9].

Although propensity score matching has been shown to improve internal validity by balancing covariates, little is known regarding the influence of sample selection on estimation that results from propensity score matching approaches [5]. Different propensity score matching procedures tend to produce a subsample that may differ from the original population, but generalizability of the results to the original sample using the matched data has rarely been examined. Although external validity is critical in contextualizing evidence for public health interventions and practices, studies using current causal inference methods, including propensity score matching, often put too little emphasis on external validity over internal validity. The purpose of this methods evaluation was to assess potential bias due to sample selection in one-to-one greedy matching as opposed to optimal full matching, which was one of the major analytic considerations in an evaluation of whether placement in a supportive housing program in New York City (NYC) reduced costs from various government services.

## Materials and Methods

### Population

In an effort to address homelessness, NYC and New York State created a program to establish 9,000 units of supportive housing for people who are homeless or at risk of homelessness in NYC. Housing placement began in 2007 and will continue until at least 2016. To evaluate the effectiveness of the program on the utilization and expenditures of government services and benefits, we conducted data linkage across multiple administrative records including other types of government housing, jails, homeless shelters, New York State psychiatric facilities, Medicaid, cash assistance, and food stamps. Data were provided by the NYC Department of Homeless Services, the NYC Department of Correction, the NYC Department of Health and Mental Hygiene, the NYC Human Resources Administration and within it Customized Assistance Services and the HIV/AIDS Services Administration, and the New York State Office of Mental Health. For the purpose of this analysis, we focused on applicants who were eligible from 2007 through 2009 for 2 of the 9 populations housed by the program. More details on the program and population definitions can be found in a recent report [10]. Readers interested in accessing the data should contact [epidatarequest@health.nyc.gov] to determine how data may be shared in a way that protects confidentiality.

One population was adults with chronic homelessness and an active substance use disorder (“SUD population”; placed: 456, unplaced: 335). The other population was young adults aging out of foster care (“young adult program”; placed: 122, unplaced: 299). The placed group included individuals who during their 1^st^ year of follow-up time were continuously placed in the supportive housing program. The unplaced group included individuals who were eligible for the program but who were not placed in the program or in any other government-subsidized housing programs tracked by the evaluation for more than 7 days [10]. The NYC Department of Health and Mental Hygiene Institutional Review Board (IRB) determined that the program evaluation is not human subject research, and therefore does not fall under the purview of the IRB.

### Variables

The exposure variable in this evaluation was living in the program for 1 year, which we refer to as being “placed,” as opposed to the comparison group that was eligible for the program but “unplaced” in it. Baseline was defined as the earliest housing placement date for the placed group and the earliest program eligibility date for the unplaced group. Among the placed group, the median difference between the first eligibility and the first placement dates was 50 days, indicating that there was not a lengthy waiting period between becoming eligible and moving into housing. This paper focuses on total Medicaid costs and Medicaid costs due to 1) outpatient care, 2) inpatient care, 3) emergency department visits, and 4) prescription drugs. We included a large number of covariates in the propensity score matching that described baseline demographic and clinical characteristics and pre-baseline service/benefit utilization (see Table S1 for the full list of covariates). We included all variables in the propensity score models except for those with extremely wide confidence intervals because those suggested multicollinearity (data about confidence intervals not shown).

### Propensity score matching

We estimated propensity scores using a logistic regression model for each population with housing placement as a dependent variable and baseline or pre-baseline covariates as independent variables. We then performed propensity score matching using 2 different algorithms. First, using a one-to-one greedy matching algorithm (i.e., nearest neighbor matching) without replacement utilizing the *MatchIt* program in R software version 2.14.2 (Vienna, Austria), we created matched pairs of placed and unplaced subjects. For the SUD population, we randomly selected one placed subject at a time and then matched that subject to an unplaced subject because the size of the placed group was larger than that of the unplaced group. For the young adult population, we used the default option in the *MatchIt* program, in which a placed subject was sequentially selected according to the largest propensity score and matched with an unplaced subject. We also performed one-to-one greedy matching using the random option and found the same matching result, confirming that matching was independent of the order of sample selection (e.g., random, largest to smallest) when the placed group was larger than the unplaced one. Second, we used the *optmatch* program in R software version 2.14.2 (Vienna, Austria) to perform optimal full matching, which generated matched sets of at least 1 placed and 1 unplaced individual as an optimal solution to minimize the total sampled distance of propensity scores. Unlike one-to-one greedy matching, optimal full matching creates matched sets that contain varying numbers of placed and unplaced subjects.

### Propensity score matching evaluation

We assessed the performance of propensity score matching using 2 criteria: 1) whether the covariates were balanced (internal validity) and 2) whether those retained in the analysis were representative of the original population included in the evaluation (external validity). For the first criterion, we evaluated the extent to which each matching method balanced differences between placed and unplaced groups by means of standardized absolute differences. Specifically, for all covariates we calculated the absolute difference in an average covariate value between placed and unplaced groups and divided that estimate by the pooled standard deviation. After incorporating propensity score matching in this calculation, we examined whether propensity score matching decreased the standardized absolute difference. If the difference became less than 0.1, which was considered to be a negligible difference in a covariate between 2 groups on average [11], we concluded that the observed covariate balance between 2 groups was achieved, and therefore propensity score matching was effective. For evaluating external validity, we compared baseline demographic and clinical characteristics and pre-baseline service/benefit utilization between the original population and the population that remained after propensity score matching, and examined whether there were systematic differences between these 2 populations by means of chi-squared tests (categorical variables) or independent t-tests (continuous variables).

### Estimation of treatment impacts

We estimated the impact of supportive housing on the difference in Medicaid costs using propensity score-matched data. After having established that covariates were balanced, which confirmed that bias due to observed confounding was unlikely and internal validity was achieved, we compared these estimates from one-to-one greedy matched data with those from optimally full-matched data, allowing us to assess potential bias due to the sample selection in a propensity score matching process (i.e., a threat to external validity). To account for skewed data and propensity score matching, we estimated median differences in outcomes by placement status by inverting the Wilcoxon signed rank test and Hodges-Lehmann (H–L) test using the one-to-one greedy-matched and the optimally full-matched data, respectively [12]. Because these 2 tests are identical in terms of their estimation algorithm (i.e., the H–L aligned rank sum test is the extension of the Wilcoxon signed rank test for matched sets), we expected to obtain almost identical point estimates if internal and external validity were established in propensity score matching mechanisms. In this study, we considered a point estimate with good internal and external validity to be a true value and assessed bias in terms of the difference between the observed and true values. Using the same tests, we tested the null hypothesis that the H–L point estimate was equal to zero.

For all analyses, statistical significance was established by a 2-sided p-value<0.05. All statistical analyses except for propensity score matching were performed using SAS 9.2 software (Cary, NC).

## Results

**Figure 1:**
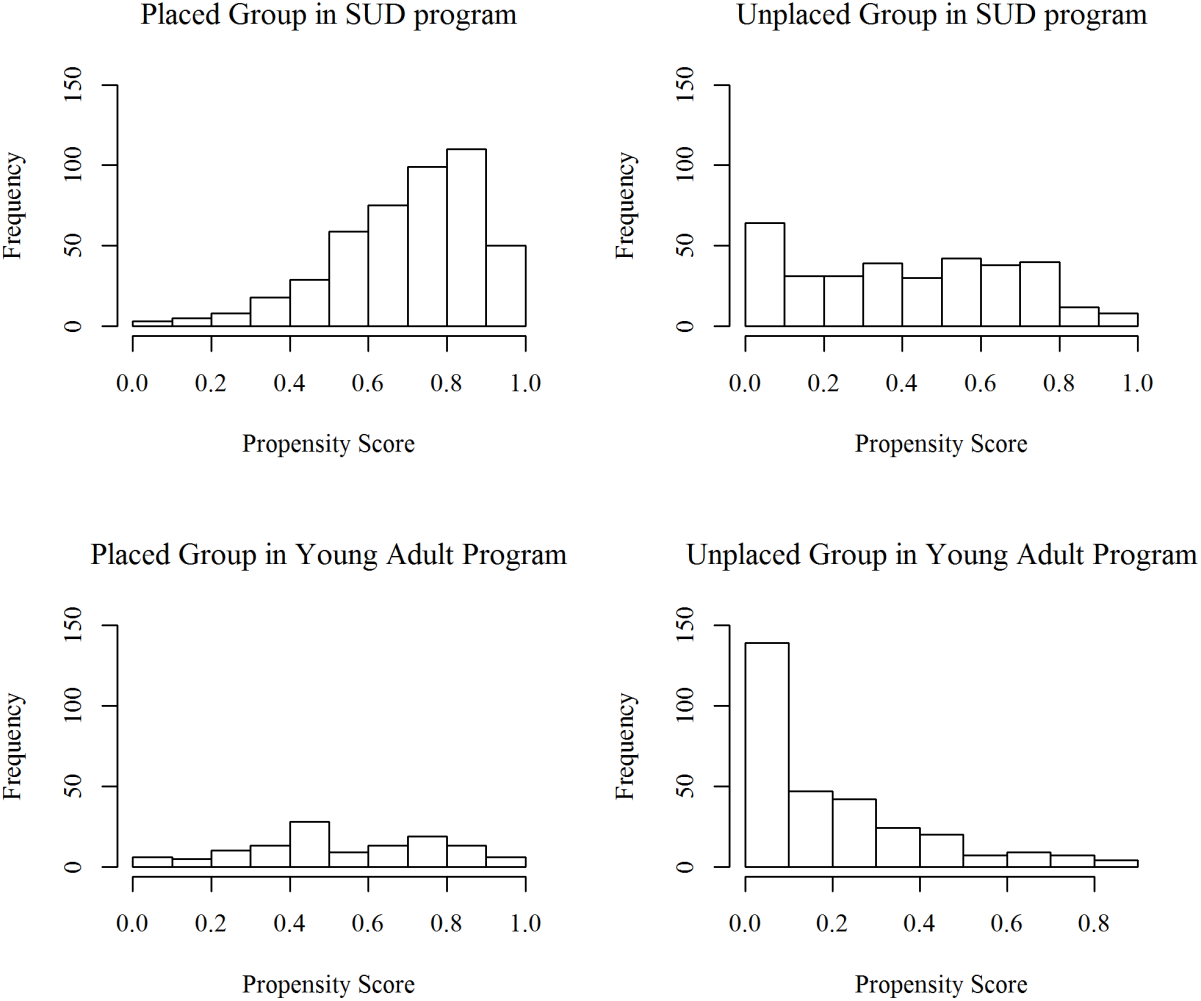
Distribution of Propensity Scores for Supportive Housing Tenants and Unplaced Applicants in Programs. This figure describes the distribution of propensity scores for placed and unplaced subjects in SUD and young adult populations. In each population, there was substantial overlap in the distributions between placed and unplaced subjects, meeting an important prerequisite for propensity score matching. *Abbreviations:* SUD, substance use disorder. Data sources: NYC Department of Homeless Services, NYC Department of Correction, NYC Department of Health and Mental Hygiene, NYC Human Resources Administration and within it Customized Assistance Services and the NYC HIV/AIDS Services Administration, and New York State Office of Mental Health.

Figure 1 describes distributions of the propensity scores (i.e., the likelihood of being continuously placed in the housing program as estimated by the propensity score models) for placed and unplaced subjects. There were substantial overlaps in the distributions between the 2 groups, meeting an important prerequisite for propensity score matching because matching placed with unplaced subjects was performed on the basis of similarities in propensity score. Stratified by propensity score quintiles, SUD program participants in lower quintile groups were more likely to be non-Hispanic white, receive supplemental security income, have mental and physical illness diagnoses, and have histories of hospitalization (data not shown). A limited capacity to live independently as measured by the number of activities of daily living requiring assistance and less frequent substance use were also associated with lower propensity score quintiles. Likewise, in the young adult population, having mental and physical illness, receiving supplemental security income, and residing in foster care or institutions such as jail or hospitals at the time of application was associated with a lower likelihood of being placed in the program.

### Balance in baseline characteristics after propensity score matching (internal validity)

Optimal full matching retained all subjects, whereas one-to-one greedy matching excluded 121 from the SUD group and 177 from the young adult group (Table 1). For the SUD program, all those excluded by one-to-one greedy matching were in the placed group and for the young adult program all were in the unplaced group. In the SUD population, the performance of one-to-one greedy matching in reducing observed differences between placed and unplaced groups greatly differed across variables, while optimal full matching in general performed well in establishing covariate balancing (Table 2). In the young adult population, both matching methods successfully reduced differences in demographic and service utilization characteristics between placed and unplaced groups.

**Table 1:** Number of supportive housing tenants and unplaced applicants in programs by propensity score quintiles.

	SUD population				Young adult population			
	Before matching		Excluded afterone-to-onematching		Before matching		Excluded after one-to-one matching	
	Placed	Unplaced	Placed	Unplaced	Placed	Unplaced	Placed	Unplaced
Total	456	335	121	0	122	299	0	177
Propensity score quintiles								
1	21	137	5	0	0	84	0	84
2	72	86	18	0	7	77	0	70
3	106	53	37	0	15	70	0	23
4	116	42	29	0	41	43	0	0
5	141	17	32	0	59	25	0	0

*Abbreviations:* SUD, substance use disorder.

Data sources: NYC Department of Homeless Services, NYC Department of Correction, NYC Department of Health and Mental Hygiene, NYC Human Resources Administration and within it Customized Assistance Services and the HIV/AIDS Services Administration, and New York State Office of Mental Health.

**Table 2:** Absolute standardized differences in selected covariates between supportive housing tenants and unplaced applicants before and after propensity score matching.

	*SUD Population*			*Young Adult Population*		
	*Before* *Matching*	*One-to-one* *Greedy* *Matching*	*Optimal* *Full* *Matching*	*Before* *Matching*	*One-to-one* *Greedy Matching*	*Optimal Full* *Matching*
US citizen	0.12	0.13	0.02	0.06	0.00	0.01
Race/ethnicity						
Non-Hispanic white	0.22	0.21	<0.01	0.14	0.17	0.23
Non-Hispanic black	0.17	0.16	0.01	0.22	0.21	0.28
Hispanic	0.01	0.01	<0.01	0.22	0.19	0.17
Other	0.06	0.05	0.01	0.12	0.09	0.07
Substance use (past)						
Never	0.06	0.05	0.00	0.39	0.16	0.07
Less than weekly	0.07	0.08	0.02	0.01	0.06	0.08
Once a week	0.03	0.02	0.03	0.09	0.11	0.03
Several times per week	0.16	0.20	0.06	0.18	0.08	0.04
Daily	0.16	0.19	0.01	0.32	0.05	0.03
Unknown	0.06	0.05	0.05	0.15	0.13	0.01
Participated in substance use treatment program	0.07	0.05	0.01	0.04	0.00	0.01
Any physical health diagnosis based on ICD-10 codes	0.21	0.17	0.06	0.23	0.09	0.09
Eligible for scattered site housing	0.54	0.52	<0.01	0.76	0.23	0.01
Total Medicaid costs‡	0.19	0.20	0.02	0.32	0.03	<0.01
Medicaid-billed inpatient costs‡	0.19	0.19	0.02	0.29	0.02	<0.01
Medicaid-billed outpatient costs‡	0.15	0.12	0.01	0.15	0.04	0.01
Medicaid-billed Emergency Department costs‡	0.26	0.23	0.01	0.19	0.02	0.01
Medicaid-billed prescription costs‡	0.10	0.09	0.04	0.12	0.02	0.01
Medicaid-billed other costs‡	0.10	0.13	<0.01	0.21	0.05	0.04

*Abbreviations:* SUD, substance use disorder.

‡ Costs were aggregated during 2 years prior to supportive housing.

Data sources: NYC Department of Homeless Services, NYC Department of Correction, NYC Department of Health and Mental Hygiene, NYC Human Resources Administration and within it Customized Assistance Services and the HIV/AIDS Services Administration, and New York State Office of Mental Health.

### Representation of the original study population after propensity score matching (external validity)

Overall there were no clear systematic differences between retained and excluded subjects in the SUD population after one-to-one greedy matching (all p>0.05 except for past violence-related symptoms/behaviors; Table 3). Even though 27% of placed subjects (n = 121) were excluded, the exclusion was independent of propensity scores, and therefore distributions were similar before and after one-to-one greedy matching. However, in the young adult population, subjects excluded by one-to-one greedy matching were predominantly from the lower quintiles, which were characterized by having mental and physical illness and current substance use, and needing assistance with activities of daily living. This resulted in systematic differences in population profiles between one-to-one greedy matched data versus the original data (Table 3). Unlike one-to-one greedy matching, optimal full matching retained all participants in both programs.

**Table 3:** Baseline characteristics and pre-supportive housing service utilization between retained and excluded subjects after one-to-one greedy matching.

	*SUD Population*				*Young Adult Population*			
	*Placed Group*			p*-value* ¶	*Unplaced Group*			p-*value* ¶
	*Total*	*Retained*	*Excluded*		*Total*	*Retained*	*Excluded*	
N	456	335	121		299	122	177	
Sex								
Female	13%	14%	9%	0.186	48%	42%	53%	0.055
Male	88%	86%	91%		52%	58%	47%	
Currently receiving supplemental security income								
Yes	20%	20%	20%	0.969	14%	3%	21%	<0.001
Current substance use based onICD-9 codes†								
Yes	85%	84%	88%	0.229	19%	12%	24%	0.013
Any physical health diagnosis basedon ICD-10 codes								
Yes	75%	74%	80%	0.159	37%	30%	41%	0.054
Any severe physical health diagnosisaccording to Charlson comorbidityindex‡								
Yes	38%	38%	41%	0.576	21%	18%	24%	0.238
Need assistance for daily living								
Yes	35%	36%	31%	0.248	51%	45%	55%	<0.001
Mental health-related symptoms/behaviors (past)								
0	70%	70%	70%	0.791	43%	61%	30%	<0.001
1	20%	20%	22%		22%	19%	24%	
2+	10%	10%	8%		35%	20%	46%	
Violence-related symptoms/behaviors(past)								
0	36%	39%	29%	0.006	28%	45%	16%	<0.001
1	48%	44%	60%		22%	22%	21%	
2+	16%	18%	11%		51%	33%	63%	
2-year service utilization prior toplacement or eligibility (average)								
Total Medicaid costs	$30,972	$30,024	$33,596	0.451	$9,202	$2112	$14,089	<0.001
Medicaid-billed outpatient costs	$5,322	$5,050	$6,074	0.256	$1,072	$544	$1,436	0.150
Medicaid-billed inpatient costs	$19,222	$19,134	$19,466	0.934	$5,858	$400	$9,620	<0.001
Cash assistance costs	$1,514	$1,548	$1,422	0.625	$335	$449	$257	0.287
Food stamp costs	$2,156	$2,185	$2,076	0.427	$744	$531	$892	0.056
Incarceration costs	$2,948	$3,038	$2,700	0.724	$1,415	$266	$2,207	0.009
Single homeless shelter costs	$15,940	$16,406	$14,650	0.322	$652	$584	$699	0.736

*Abbreviations:* SUD, substance use disorder.

† ICD-9 codes include 29100,29110,29120,29130,29140,29150,29180,2911,2912,2915, 2919,29181,29182,29189,29190,30300,3030,303,30301,30302,30303,30390, 3039,30391,30392,30393,30500,305,3050,30501,30502,30503,76071,9800,29200,29211,29212,29220,29281,29282,2929, 29283,29284,29285,29289,29290,30400,304,3040,30401,30402,30403, 30410,3041,30411,30412,30413,30420,3042,30421,30422,30423,30430,3043, 30431,30432,30433,30440,3044,30441,30442,30443,30450,3045,30451, 30452,30453,30460,3046,30461,30462,30463,30470,3047,30471,30472, 30473,30480,3048,30481,30482,30483,30490,3049,30491,30492,30493,30520,3052,30521,30522,30523,30530,3053,30531,30532,

30533,30540,3054,30541,30542,30543,30550,3055,30551,30552,30553,30560,3056,30561,30562,30563,30570,30571,30572,

30573,30580,3058,30581,30582,30583,30590,3059,30591,30592,30593,64830,64831,64832,64833,64834,65550,65551,65553, 76072,76073,76075,77950,96500,9650,96501,96502,96509,V6542. These codes were informed by Healthcare Cost and Utilization Project (www.hcup-us.ahrq.gov/toolssoftware/ccs/ccs.jsp).

‡ ICD-10 codes include I21, I22, I25, I43, I50, I09, I11, I13, I42, P29, I70,I71,I73,I77,I79,K55,Z95,G45,G46,I60,I61,I62,I63, I64,I65,I66,I67,I68,I69,H34,F00,F01,F02,F03,G30,F05,G31,J40,J41,J42,J43,J44,J45,J46,J47,J60,J61,J62,J63,

J64,J65,J66,J67,I27,J68,J70,M05,M32,M33,M34,M06,M31,M35,M36, K25,K26,K27,K28,B18,K73,K74,K70,K71,K76,Z94,

E10,E11,E12,E13,E14,G81,G82,G04,G11,G80,G83, N18,N19,N05,N25,I12,I13,N03,Z49,Z94,Z99,C00,C01,C02,C03,C04,

C05,C06,C07,C08,C09,C10,C11,C12,C13,C14,C15,C16,C17,C18,C19, C20,C21,C22,C23,C24,C25,C26,C30,C31,C32,C33,C34,C37,C38,C39, C40,C41,C43,C45,C46,C47,C48,C49,C50,C51,C52,C53,C54, C55,C56,C57,C58,C60,C61,C62,C63,C64,C65,C66,C67, C68,C69,C70,C71,C72,C73,C74,C75,C76,C81,C82,C83,C84,C85,C88,C90,C91,C92,C93,C94,C95,C96,C97,K70,K71,K72,K76,

I85,I86,I98,C77,C78,C79,C80,B20,B21,B22,B23,B24. Charlson ME, Pompei P, Ales KL, MacKenzie CR (1987) A new method of classifying prognostic comorbidity in longitudinal studies: development and validation. J Chron Dis 40(5): 373–383.

¶ *p*-values were derived from chi-squared tests (categorical variables) or independent *t*-tests.

Data sources: NYC Department of Homeless Services, NYC Department of Correction, NYC Department of Health and Mental Hygiene, NYC Human Resources Administration and within it Customized Assistance Services and the HIV/AIDS Services Administration, and New York State Office of Mental Health.

### Estimated differences in outcomes associated with treatment

Given good internal and external validity, we considered the estimated program impact from optimally full-matched data to be a gold standard, and compared it with estimates using one-to-one greedy matched data to assess bias. For the SUD population the estimated program impacts on Medicaid costs were generally greater using one-to-one greedy matching as opposed to optimal full matching (Table 4). In contrast, for the young adult population the estimated program impacts on total Medicaid costs and outpatient Medicaid costs were attenuated when one-to-one greedy matching versus optimal full matching was used. Given similar covariate distributions for one-to-one greedy and optimally full-matched data in the SUD population, which indicated external validity, the discrepancies in estimates were likely due to covariate imbalances (i.e., low internal validity) that one-to-one greedy matching failed to reduce. In the young adult population, given that both matching methods effectively established internal validity, the differences were more likely to be attributed to the one-to-one greedy matching process that systematically excluded people with low propensity scores who were likely to experience a greater impact of the supportive housing program on total Medicaid costs given their baseline characteristics (i.e., low external validity).

**Table 4:** Estimated program impacts† on one-year Medicaid costs post supportive housing using optimal full matching versus one-to-one greedy matching.

	*SUD Population*				*Young Adult Population*			
*One-year Medicaid* *cost post-supportive* *housing*	*Optimal Full* *Matching*		*One-to-one Greedy* *Matching* ¶		*Optimal Full Matching*		*One-to-one Greedy* *Matching*	
	*Point* *Estimate*	*p-value*	*Point* *Estimate*	*p-value*	*Point* *Estimate*	*p-value*	*Point Estimate*	*p-value*
Total Medicaid costs	−$3,600	0.003	−$5,097	<0.001	−$580	0.137	−$272	0.463
Medicaid inpatientcosts	−$1,330	0.010	−$7,414	<0.001	NE‡		NE‡	
Medicaid outpatientcosts	−$330	0.188	−$74	0.763	$30	0.446	−$3	0.981
Medicaid emergencydepartment costs	−$108	0.001	−$387	<0.001	$0	0.840	$3	0.935
Medicaid prescriptiondrug costs	−$97	0.224	−$91	0.198	−$2	0.736	−$4	0.827

*Abbreviations:* NE, non-estimable; SUD, substance use disorder.

† These estimates were based on Hodges-Lehmann (full matching) and Wilcoxon (one-to-one matching) signed rank test (two-sided *p*-value).

‡ Some estimates were non-estimable because a majority of subjects had zero outcomes.

¶ Because there were more placed subjects than unplaced ones, each random selection of placed subjects prior to matching produced slightly different matched pairs, which resulted in slightly different estimates.

Data sources: NYC Department of Homeless Services, NYC Department of Correction, NYC Department of Health and Mental Hygiene, NYC Human Resources Administration and within it Customized Assistance Services and the HIV/AIDS Services Administration, and New York State Office of Mental Health.

## Discussion

In this evaluation we demonstrated that one-to-one greedy matching led to biased estimates when selection was not independent of the program impact. In the young adult population, one-to-one greedy matching systematically excluded unplaced subjects with low propensity scores, generating a matched population that was healthier and more independent in daily living than the original one. Despite good internal validity, estimated program effectiveness was attenuated compared with that from optimally full-matched data. In contrast, for the SUD population the sample selection for one-to-one greedy matching appeared to be independent of propensity scores, indicating external validity. For this population some differences in the program impact between the two matching methods were observed, which was likely due to covariate imbalance, rather than selection bias.

Current literature offers little discussion of influences on estimation due to sample selection with propensity score matching mechanisms. This may be because this potential selection bias does not occur when a treatment impact is estimated only for the treatment group [13]. Yet, in some contexts where understanding the impact of a treatment among the entire population of interest is desired, selecting subjects for propensity score matching could introduce unintended bias into estimation. Such a case would be the evaluation of a public health intervention targeted to a particular population, e.g., what change in outcomes would have occurred if all subjects in the population had received a treatment?

Our findings support Little and Rubin’s argument that if sample selection is non-ignorable, the size of bias in the estimated population-level effects depends on the degree of association between treatment effects and selection after adjusting for covariates [8], [9]. We found that sample selection in one-to-one greedy matching depended on the extent to which the propensity score distribution overlapped between placed and unplaced groups and the sample size of these groups. In addition, our findings confirmed current evidence that optimal full matching that employs flexible matching ratios is more effective in covariate balancing than one-to-one greedy matching [14]. Despite the advantage of optimal full matching over one-to-one greedy matching in establishing both internal and external validity, one-to-one greedy matching tends to be a popular propensity score matching choice because analysis of matched pairs and interpretation of the results are more conceptually and computationally straightforward than those of optimal full matching. With limited emphasis on potential selection bias, researchers often justify using one-to-one greedy matching if covariate balancing is observed. Our findings highlight the importance of examining both internal and external validity in determining a propensity score matching method.

There are some limitations to this evaluation. First, we have not identified variables that are a common effect of treatment and outcome (collider) or located in the causal pathway from treatment and outcome (mediator) among covariates. Estimates could be biased due to controlling for these variables via propensity score matching [15]. To minimize this potential distortion of true association between treatment and outcome (e.g., biased either away or toward to the null), we only used baseline and pre-baseline covariates. Second, unobserved covariates could have biased estimates. However, the study focused on differences between 2 propensity score matching methods using the same data and differential influences from unobserved covariates by matching methods were quite unlikely. Despite these limitations, a main strength of this evaluation includes the well-defined comparison group that consists of applicants eligible for the housing program. Another strength is that multiple administrative data sources provided a large number of baseline and pre-baseline characteristics, which improved the estimation of propensity scores.

Propensity score matching is a useful tool to reduce bias due to confounding and estimate a treatment effect when there is sufficient overlap in the distribution of propensity scores between treatment and control groups. Yet, unintended selection bias can arise when sub-setting the original population for matching is associated with program impact. In this evaluation, we provide a practical diagnostic approach to assessing potential selection bias in propensity score matching mechanisms that we used in a program evaluation. When inference is made to the whole study population in a program evaluation, we suggest considering optimal full matching over one-to-one greedy matching to strengthen both internal and external validity and minimize potential selection bias.

## Supporting Information

Table S1 **Covariates included in the propensity score models**. This table lists all the covariates that we included in the propensity score models.
(DOCX)

Text S1 **This text includes R codes that allow for performing two types of propensity score matching (optimal full matching and one-to-one greedy matching) and the Wilcoxon signed rank test.** It also includes SAS codes that can be be used for performing the Hodges-Lehmann aligned rank sum test and for obtaining Hodges-Lehmann estimators.
(DOCX)
