# Supplementary material for: Bias Due to Sample Selection in Propensity Score Matching for a Supportive Housing Program Evaluation in New York City
Source: PLoS One. 2014 Oct 13;9(10):e109112. doi: 10.1371/journal.pone.0109112 (PMC4195658; doi:10.1371/journal.pone.0109112)
Supplement: Table S1 — Covariates included in the propensity score models. This table lists all the covariates that we included in the propensity score models. (DOCX) [file pone.0109112.s001.docx]

Table S1. Covariates included in the propensity score models

|  | Covariates | SUD population | Young adult population |
| --- | --- | --- | --- |
| Baseline demographic characteristics | Age on 1/1/2007 (categories) |  |  |
|  | Sex |  |  |
|  | Race/ethnicity |  |  |
|  | Education |  |  |
|  | Citizenship |  |  |
|  | Language |  |  |
|  | Veteran status |  |  |
| Baseline substance use characteristics | Current substance use pattern |  |  |
|  | Past substance use pattern |  |  |
|  | Currently participating in substance use program |  |  |
|  | Completed substance use program |  |  |
|  | Diagnosed with substance use disorders |  |  |
| Baseline mental health characteristics | Diagnosed with mental illness or substance use disorders |  |  |
|  | Has mental retardation |  |  |
|  | Has any mental illness other than mental retardation |  |  |
|  | History of mental health-related symptoms/behaviors |  |  |
|  | Current mental health-related symptoms/behaviors |  |  |
|  | History of violent symptoms/behaviors |  |  |
|  | Current violent symptoms/behaviors |  |  |
|  | Past psychiatric hospitalization |  |  |
| Baseline physical health characteristics | Number of types of physical illness diagnoses (categories) |  |  |
|  | Comorbidity of mental and physical diagnoses |  |  |
|  | Comorbidity of mental and severe physical diagnoses |  |  |
|  | Number of activities of daily living that require assistance (categories) |  |  |
|  | Currently hospitalized |  |  |
|  | Currently hospitalized, incarcerated, in foster care, or in other institution |  |  |
| Housing eligibility information | Approved for single-site housing |  |  |
|  | Approved for scattered site housing |  |  |
|  | Recommended for 24-hour supervision |  |  |
|  | Recommended for assisted outpatient treatment |  |  |
|  | Recommended for case management |  |  |
|  | Recommended for child care services |  |  |
|  | Recommended for domestic violence services |  |  |
|  | Recommended for financial management services |  |  |
|  | Recommended for primary health care |  |  |
|  | Recommended for vocational/educational training program |  |  |
|  | Recommended for medical treatment |  |  |
|  | Recommended for medication management |  |  |
|  | Recommended for mental health treatment |  |  |
|  | Recommended for Mental illness and Chemical Addition program |  |  |
|  | Recommended for parenting skills training |  |  |
|  | Recommended for psychiatric treatment |  |  |
|  | Recommended for substance use treatment |  |  |
| Receipt of benefits at baseline | Social security |  |  |
|  | Supplementary Security Income |  |  |
|  | HIV/AIDS Services Administration |  |  |
|  | Pension |  |  |
|  | Medicare |  |  |
|  | Veteran's benefits |  |  |
| Amount of services/benefits received during 2 years prior to baseline | Days in New York City single adult homeless^‡^ shelters |  |  |
|  | Days in New York City family homeless^‡^ shelters |  |  |
|  | Days in New York City jails^‡^ |  |  |
|  | Costs of Medicaid emergency department visits^‡^ |  |  |
|  | Costs of Medicaid inpatient^‡^ hospitalizations |  |  |
|  | Costs of Medicaid outpatient visits^‡^ |  |  |
|  | Costs of Medicaid-reimbursed prescriptions^‡^ |  |  |
|  | Costs of Medicaid due to other reasons^‡^ |  |  |
|  | Costs from food stamps^‡^ |  |  |
|  | Costs from cash assistance^‡^ |  |  |
|  | Total services/benefits costs^‡^ |  |  |

Abbreviations: NE, non-estimable; SUD, Substance Use Disorder.

Variables checked were included in the propensity score models. Unchecked variables were not included because of multicollinearity issues.

^‡^continuous variables.

Data sources: NYC Department of Homeless Services, NYC Department of Correction, NYC Department of Health and Mental Hygiene, NYC Human Resources Administration and within it Customized Assistance Services and the HIV/AIDS Services Administration, and New York State Office of Mental Health.
