# Supplementary material for: Bias Due to Sample Selection in Propensity Score Matching for a Supportive Housing Program Evaluation in New York City
Source: PLoS One. 2014 Oct 13;9(10):e109112. doi: 10.1371/journal.pone.0109112 (PMC4195658; doi:10.1371/journal.pone.0109112)
Supplement: Text S1 — This text includes R codes that allow for performing two types of propensity score matching (optimal full matching and one-to-one greedy matching) and the Wilcoxon signed rank test. It also includes SAS codes that can be be used for performing the Hodges-Lehmann aligned rank sum test and for obtaining Hodges-Lehmann estimators. (DOCX) [file pone.0109112.s002.docx]

#SUPPORTING INFORMATION TEXT S1

#Author: Sungwoo Lim

#To run propensity score matching, the author adopted R codes from optmatch #(http://cran.r-project.org/web/packages/optmatch/optmatch.pdf) and MatchIt #packages manuals (http://cran.r-#project.org/web/packages/MatchIt/MatchIt.pdf).

#To perform Hodges-Lehmann tests and compute Hodges-Lehmann point estimates, #the author wrote SAS macros based on “Rosenbuam PR (2010) Design of #Observational Studies. Springer Series in Statistics” and “hodgesl” (stata #module to perform Hodges-Lehmann aligned rank test; #http://fmwww.bc.edu/repec/bocode/h/hodgesl.ado)

#The following R codes were used to perform optimal full matching and one-to-#one greedy matching

#Please replace id, tx (treatment), and prob (propensity scores) with #corresponding variables in your data

**R codes**

library(optmatch)

a<-read.csv("data.csv")

#Optimal full matching

scalardiffs <-function(trtvar,data,scalarname) {

sclr<-data[names(trtvar),scalarname]

names(sclr)<-names(trtvar)

abs(outer(sclr[trtvar],sclr[!trtvar], '-'))

}

psd2<-makedist(tx~1, a, scalardiffs, "prob")

bbb<-fullmatch(psd2,data=a)

stratumStructure(bbb)

b<-data.frame(bbb)

b<-cbind(a$id,b)

colnames(b)<-c("id","strata")

# pair matching

library(MatchIt)

pair.out<-matchit(tx~ LIST COVARIATES FROM YOUR DATA

, data=a,method="nearest",m.order="random")

pair.tx<-match.data(pair.out,group="treat",subclass="pair")

pair.ct<-match.data(pair.out,group="control",subclass="pair")

pair.tx$id <-1:nrow(pair.tx)

pair.ct$id <-1:nrow(pair.ct)

# The following R codes were used to obtain p-values of treatment effects #using one-to-one matched data

#Wilcoxon signed rank test

wilcox.test(pair.tx$NAME OF TREATMENT VARIABLE, pair.ct$NAME OF TREATMENT VARIABLE, paired=TRUE, estimate=TRUE,conf.int=TRUE)

####Repeat with all of the treatment variables###

wilcox.test(pair.tx$outcome, pair.ct$outcome, paired=TRUE,

/*The following SAS macro was used to perform the Hodges-Lehmann test to test whether the*/

/*treatment effect was statistically significant*/

/*If you want to use this macro for your program evaluation, please do the following: */

/*1. Run optimal full matching*/

/*2. Execute the macro after specifying variables*/

/*%hl(data=your data, outcome = your outcome, tx = your treatment, id = your id) */

**SAS codes**

/*Hodges-Lehmann test */

**%macro** hl (data,outcome,tx,id);

data temp;

set &data;

y = &outcome;

run;

/*we need to count the number of observations in the dataset,

which HL needs to do its analysis*/

proc sql;

select count(&id) into: total

from temp

where strata ~= **.**;

quit;

/*1. create mean outcome by strata (r1)*/

proc sql;

create table r1 as

select strata, mean(&outcome) as m_y

from temp

group by strata;

quit;

/*2. create stratum-specific weight (r2)*/

proc sql;

create table r2 as

select strata, &tx, count(&outcome) as m_n

from temp

group by strata, &tx;

quit;

data r2 (keep = strata factor ni);

set r2;

if &tx = **0** then mi = m_n;

if &tx = **1** then ni = m_n;

mii = lag(mi);

n = ni+lag(mi);

factor = (mii*ni)/(n*(n-**1**));

if factor = **.** then delete;

run;

/*3. center the outcome*/

proc sql;

create table r0 as

select a.strata, a.&tx, a.&outcome, b.m_y

from temp as a left join r1 as b

on a.strata=b.strata;

quit;

data r0;

set r0;

dy = &outcome - m_y;

run;

/*4. create a dataset 'rank' with a variable 'rk' ranking 'dy' in ascending order*/

proc sort data =r0;

by dy;

run;

proc rank data=r0 out= rank (rename = (dy = rk)) ;

var dy;

run;

data rank (keep = strata dy rk &tx);

set rank;

dy = &outcome - m_y;

run;

/*5. create a sum of ranking variable (wsi) by strata only for the treatment group */

proc sql;

create table r3 as

select strata, sum(rk) as wsi

from rank

where &tx = **1**

group by strata;

quit;

/*6. create a mean ranking variable (ki) by strata*/

proc sql;

create table r4 as

select strata, mean(rk) as ki

from rank

group by strata;

quit;

/*7. create an expected ranking variable (e_wsi = ki*ni)by strata for the treatment group only*/

proc sort data=r2;

by strata;

proc sort data=r4;

by strata;

data r4;

merge r4 (in=a1) r2 (in=a2);

by strata;

e_wsi = ki*ni;

run;

/*8. Merge r3 with r4 and calculate k = (rk - ki)^2 for each stratum*/

/* Then, create the sum of k (ss_kd) by strata*/

proc sort data=rank;

by strata;

proc sort data=r4;

by strata;

data r5;

merge r4 (in=a1) rank (in=a2);

by strata;

k = (rk-ki)*(rk-ki);

run;

proc sql;

create table r6 as

select strata, sum(k) as ss_kd

from r5

group by strata;

quit;

/*9. combine all pieces*/

proc sort data=r2; by strata; /*factor (=weight)*/

proc sort data=r3; by strata; /*wsi ( = sum of ranking for treatment)*/

proc sort data=r4; by strata; /*e_wsi (= expected ranking for treatment)*/

proc sort data=r6; by strata; /*ss_kd (= sum of variance)*/

data r6;

merge r2 r3 r4 r6;

by strata;

var_wsi = factor*ss_kd;

run;

/*10. finally compute test statistics to derive two-tailed p-values of testing whether the difference between the treatment and control groups is

statistically significant*/

proc sql;

create table h_l as

select sum(wsi) as ws, sum(e_wsi) as e_ws, sum(var_wsi) as var

from r6;

quit;

data h_l;

set h_l;

z = (ws-e_ws)/sqrt(var);

p = **2***(**1**-cdf('normal',abs(z),**0**,**1**));

format p **9.5**;

run;

proc print data=h_l noobs;

title "&outcome";

var z p;

run;

**%mend**;

/*In the following SAS macro, you can obtain a Hodges-Lehmann estimator*/

/*If you want to use this macro for your program evaluation, please do the following: */

/*1. Run optimal full matching */

/*2. Execute the macro after specifying variables*/

/*assuming that a treatment effect is between 0 and 100*/

/*%hl2(data=your data, outcome = your outcome, tx = your treatment, id = your id, min=0, max= 100,by=10)*/

/*Hodges-Lehmann estimator */

**%macro** hl2(data,outcome,tx, id, min, max, by);

%local i;

%do i = **1** %To %SysFunc( Ceil( %SysEvalF( ( &max - &min ) / &by ) ) ) ;

%let value=%SysEvalF( ( &min - &by ) + ( &by * &i ) ) ;

%put &value;

data p (keep = strata y &tx);

set &data;

if &tx = **1** then y = &outcome-&value;

else y = &outcome;

run;

/*1. create mean outcome by strata (r1)*/

proc sql;

create table r1 as

select strata, mean(y) as m_y

from p

group by strata;

quit;

/*2. create stratum-specific weight (r2)*/

proc sql;

create table r2 as

select strata, &tx, count(y) as m_n

from p

group by strata, &tx;

quit;

data r2 (keep = strata factor ni);

set r2;

if &tx = **0** then mi = m_n;

if &tx = **1** then ni = m_n;

mii = lag(mi);

n = ni+lag(mi);

factor = (mii*ni)/(n*(n-**1**));

if factor = **.** then delete;

run;

/*3. center the outcome*/

proc sql;

create table r0 as

select a.strata, a.&tx, a.y, b.m_y

from p as a left join r1 as b

on a.strata=b.strata;

quit;

data r0;

set r0;

dy = y - m_y;

run;

/*4. create a dataset 'rank' with a variable 'rk' ranking 'dy' in ascending order*/

proc sort data =r0;

by dy;

run;

proc rank data=r0 out= rank (rename = (dy = rk)) ;

var dy;

run;

data rank (keep = strata dy rk &tx);

set rank;

dy = y - m_y;

run;

/*5. create a sum of ranking variable (wsi) by strata only for treatment group */

proc sql;

create table r3 as

select strata, sum(rk) as wsi

from rank

where &tx = **1**

group by strata;

quit;

/*6. create a mean ranking variable (ki) by strata*/

proc sql;

create table r4 as

select strata, mean(rk) as ki

from rank

group by strata;

quit;

/*7. create an expected ranking variable (e_wsi = ki*ni)by strata for treatment only*/

proc sort data=r2;

by strata;

proc sort data=r4;

by strata;

data r4;

merge r4 (in=a1) r2 (in=a2);

by strata;

e_wsi = ki*ni;

run;

/*8. Merge r3 with r4 and calculate k = (rk - ki)^2 for each stratum*/

/* Then, create sum of k (ss_kd) by strata*/

proc sort data=rank;

by strata;

proc sort data=r4;

by strata;

data r5;

merge r4 (in=a1) rank (in=a2);

by strata;

k = (rk-ki)*(rk-ki);

run;

proc sql;

create table r6 as

select strata, sum(k) as ss_kd

from r5

group by strata;

quit;

/*9. combine all pieces*/

proc sort data=r2; by strata; /*factor (=weight)*/

proc sort data=r3; by strata; /*wsi ( = sum of ranking for treatment)*/

proc sort data=r4; by strata; /*e_wsi (= expected ranking for treatment)*/

proc sort data=r6; by strata; /*ss_kd (= sum of variance)*/

data r6;

merge r2 r3 r4 r6;

by strata;

var_wsi = factor*ss_kd;

run;

/*10. finally compute test statistics to derive two-tailed p-values of testing whether the difference between the treatment and control groups is

statistically significant*/

proc sql;

create table h_l as

select sum(wsi) as ws, sum(e_wsi) as e_ws, sum(var_wsi) as var

from r6;

quit;

data p2 (keep = x p1);

set h_l;

x = &value;

z = (ws-e_ws)/sqrt(var);

p1 = **1**-cdf('normal',abs(z),**0**,**1**);

format p1 **9.5**;

run;

proc datasets nolist;

delete r0 r1 r2 r3 r4 r5 r6 rank;

run;

quit;

%if &i = **1** %then %do;

data min&outcome;

set p2;

run;

%end;

%else %do;

proc append base=min&outcome data=p2;

run;

proc delete data=p2;

run;

%end;

%end;

**%mend**;
